# Dynamic Packet Duplication for Industrial URLLC

**DOI:** 10.3390/s22020587

**Published:** 2022-01-13

**Authors:** David Segura, Emil J. Khatib, Raquel Barco

**Affiliations:** Instituto Universitario de Investigación en Telecomunicación (TELMA), Universidad de Málaga, CEI Andalucía TECH E.T.S.I. Telecomunicación, Bulevar Louis Pasteur 35, 29010 Malaga, Spain; emil@uma.es (E.J.K.); rbm@ic.uma.es (R.B.)

**Keywords:** 5G, machine learning, prediction, Industry 4.0, URLLC, multi-connectivity

## Abstract

The fifth-generation (5G) network is presented as one of the main options for Industry 4.0 connectivity. To comply with critical messages, 5G offers the Ultra-Reliable and Low latency Communications (URLLC) service category with a millisecond end-to-end delay and reduced probability of failure. There are several approaches to achieve these requirements; however, these come at a cost in terms of redundancy, particularly the solutions based on multi-connectivity, such as Packet Duplication (PD). Specifically, this paper proposes a Machine Learning (ML) method to predict whether PD is required at a specific data transmission to successfully send a URLLC message. This paper is focused on reducing the resource usage with respect to pure static PD. The concept was evaluated on a 5G simulator, comparing between single connection, static PD and PD with the proposed prediction model. The evaluation results show that the prediction model reduced the number of packets sent with PD by 81% while maintaining the same level of latency as a static PD technique, which derives from a more efficient usage of the network resources.

## 1. Introduction

Wired communications have been widely used in industrial scenarios as new applications of automation and Artificial Intelligence (AI) with high mobility are rolled out. However, wired communications are costly in terms of installation and maintenance and cannot cover new use cases, such as mobility in factories. As a result, the whole industry is shifting towards more flexible and adaptable scenarios, resulting in the Industry 4.0 paradigm. Industry 4.0 is the fourth industrial revolution to improve the flexibility of production and distribution processes where wireless networks have an important part.

The advances in the fields of robotics, AI and Machine Learning (ML) converge in Industry 4.0 to adapt production to new customer demands, such as an increased customization, reduced costs and lower environmental impact [1]. Wireless networks are a major enabler of flexibility in Industry 4.0, allowing easy reconfiguration of production lines, mobile appliances, such as robots both within and beyond the bounds of factories.

The fifth-generation (5G) radio technology, which has been standardized by the 3rd Generation Partnership Project (3GPP) members, aims to provide more flexibility to support new services and applications. Unlike previous technologies, that were focused on traditional mobile broadband, in 5G new services categories have been defined according to their requirements:

- Enhanced Mobile BroadBand (eMBB): this service category is an evolution of traditional mobile broadband, with higher data rates (up to 20 Gbps) and bandwidth. It is similar to the traditional use of networks by users, such as web browsing or streaming multimedia content.
- Massive Machine-Type Communications (mMTC): this service category covers massive connection of devices, with a sporadic and lower volume of data exchange over the network. It is mainly focused on the Internet of Things (IoT).
- Ultra-Reliable and Low Latency Communications (URLLC): this service category aims to cover critical communications, where short messages are exchanged with requirements of lower latency and higher reliability. The latency requirement varies from 1 to 15 ms, depending on the application itself. However, in 5G, it is expected to reach a maximum latency of 1 ms with a reliability target of $1-10^{-5}$ for a packet size of 32 bytes at the user plane [2].

URLLC can support use cases, such as closed loop control, hazard sensors, robot automatization, augmented/virtual reality, drone communications and mobile eHealth. The latency requirement varies from 1 to 15 ms, while the reliability targets can range between $1-10^{-5}$ and $1-10^{-9}$ [3]. These use cases need advanced radio features and resource cost techniques to fulfill highly demanding latency and reliability targets. There are several approaches to achieve such requirements. One technique is the reduction of the time-slot duration by means of a higher numerology [4,5] and by changing the radio resource scheduler [6].

Another solution consists of eliminating steps in the connection protocols to reduce the access time—known as Grant-free transmission [7]. Multi-connectivity [8,9] has been proposed for the sake of achieving high reliability and low latency. In particular, for the sake of achieving high reliability, many solutions have been proposed. The studies in [9,10] are focused on a static threshold (i.e., Reference Signal Received Power (RSRP) or Channel Quality Indicator (CQI)) that determines the dual connectivity range or Packet Duplication (PD) activation.

In [11], eMBB/URLLC multiplexing through preemptive URLLC puncturing was studied, where a Deep Learning Link Adaptation was proposed for eMBB users to maximize the throughput, while ensuring reliability for eMBB users due to corruption of the packets derived from URLLC puncturing. In [12], the PD architecture for carrier aggregation and the dual connectivity approach is presented. The study focuses on how many links are necessary to fulfill reliability and latency requirements for URLLC. A conservative link adaptation algorithm for URLLC is proposed in [13], where the base station keeps statistics of received CQI reports and calculates the maximum channel quality degradation over a time window.

Moreover, in [14] increasing the number of CQI and Modulation Coding Scheme (MCS) values is proposed to reduce the gap between the Signal to Interference plus Noise Ratio (SINR) threshold values and, hence, the radio resource wastage. In [15], two methods for enhancing the efficiency of data duplication over dual connectivity are proposed. In the first method, the duplicates of packets already successfully delivered to the User Equipment (UE) are promptly dropped from the transmission queues of the involved base stations using an uplink indication provided by the UE.

In the second method, the duplication is only performed when the primary base station receives a negative acknowledgement (NACK) associated with the transmission. A Deep Reinforcement Learning method was proposed in [16] to decide which secondary legs to use to duplicate and transmit the packet for the UE within the dual connectivity range. The studies above specifically cover Urban Macro (UMa) and Urban Micro (UMi) scenarios, but not the industrial scenario.

Although these techniques have shown their effectiveness in reducing latency and packet loss, they come at an additional cost, which derives from a less efficient usage of the network resources. The usage of these techniques does not add any benefit if the baseline network can guarantee the requirements of the services/applications at a given moment. Prediction may allow the base station to decide if a redundant technique is required.

This paper proposes a method to predict the End-to-End (E2E) latency as a Service Key Performance Indicator (S-KPI) [17] by observing the network conditions right before a critical transmission in the downlink and, based on that prediction, activate the PD technique (dynamic algorithm) [12,18] in industrial scenarios. The approach for the prediction model followed in this paper is based on the solution shown in [19], where Radio Access Network (RAN) Key Performance Indicators (KPIs), such as RSRP and Reference Signal Received Quality (RSRQ), are used to predict the S-KPIs using an estimator based on ML.

In particular, the authors in [19] applied the estimator for video Key Quality Indicators (KQIs), such as the average buffer size or number of stalls. Nevertheless, the methodology is independent to the final application and ML algorithm used. In this paper, the same concept is adopted for the prediction of the E2E latency S-KPI, assuming that the main contribution of the latency is at the RAN level. As in 5G, it is expected that Mobile Edge Computing (MEC) [20] will play a central role for URLLC, this assumption is justifiable, since the path of the packets will be limited to the RAN.

The advantage of this approach is that RAN KPIs can be easily measured by the network terminals and infrastructure elements. In this paper, the precision of the estimator is evaluated. The dynamic PD approach using the predictor was evaluated, comparing the E2E latency performance with single connection and a static PD (that is, always duplicating the packet regardless of network conditions) techniques and the resource consumption when using a static vs. a dynamic PD approach (based on the proposed latency predictor).

The remainder of this paper is organized as follows. In Section 2, the materials are described: first, a brief description of cellular networks in Industry 4.0 and critical applications is given in Section 2.1; and in Section 2.2, multi-connectivity in 5G is presented. Then, in Section 3, the proposed system is described. In Section 4, the simulated implementation details are described. The results are shown in Section 5. Finally, our conclusions are drawn in Section 6.

## 2. Background

### 2.1. Industrial Networks

#### 2.1.1. Wireless Connectivity in Industry

Wired connections have been widely used in industrial networks, such as ProfiNET, EtherCAT and the set of Time Sensitive Networks (TSN) protocols. Nevertheless, wired infrastructures are costly in terms of installation and maintenance. These are also not suitable for novel Industry 4.0 use cases, such as mobile robots.

In wireless technologies, there is a division between two types of networks that enables different applications. The division is regarding Local Area Networks (LAN) and Wide Area Networks (WAN). LANs have a coverage range of up to 100 m; covering areas, such as rooms or even full factories. The main wireless LAN technologies are based on the IEEE 802.11 family—commonly named WiFi. Moreover, there exist customized solutions for factories, based on IEEE 802.15.1 and 802.15.4, such as Wireless Interface to Sensors and Actuators (WISA) and WirelessHART. These technologies operate in an unlicensed spectrum and suffer from poor scalability [21].

On the other hand, WANs provide a higher coverage range, from distances of a few kilometers up to whole countries. The most extended and known wireless WANs are the 3GPP-based technologies (GSM, GPRS, EDGE, UMTS, LTE and 5G). As cellular networks operate at a licensed spectrum, a better performance can be obtained. Cellular networks provide ubiquitous connectivity beyond the limits of the factory.

This means that production that is geographically distributed can be monitored and managed using a single network. As WANs may be provided as a service by cellular network operators, they do not imply the acquisition, installation and maintenance of infrastructure, allowing multi-tenancy and, therefore, resulting in lower costs [22] and improved connectivity.

Wireless networks generally suffer from a higher latency and packet loss probability than wired networks. For this reason, the recent generations of cellular networks have introduced different optimizations for reducing the latency and also improving reliability.

For 5G, Industry 4.0 is one of the main development verticals, where its applications have been explored for a special network design. One of the main novelties in 5G is the introduction of MEC [20]. MEC consists of moving the application servers to the network edge, thus, reducing the path that a packet must travel. This approach reduces the latency for the devices and the network load beyond the RAN.

#### 2.1.2. Critical Applications in Industry 4.0

In the Industry 4.0 paradigm, agility is a key objective in the design of factories. Agility means the flexibility of the system to changing requirements by replacing or improving separated modules. Some of the main technologies that allow such agility in factories are the following:

- Rearrangeable modules in production lines [23]: traditionally, production lines have been made up of static modules that perform specific operations. These modules, each controlled by a Programmable Logic Controller (PLC), are interconnected via wired to the Manufacturing Execution System (MES). By enabling the mobility of these modules, new combinations of elements into new types of production lines are possible.
- Automated Guided Vehicle (AGV) [24]: it is common that vehicles driven by workers perform tasks, such as moving stocks and supplies in factories. In the Industry 4.0 paradigm, due to the customization of production, these kinds of movements increase exponentially. It is harder to provide supplies in batches; therefore, smaller vehicles are required with an increase in the number and variety of trips. To achieve this without increasing the workload, AGVs do this without the need for human drivers.
- Drones [25]: drones are a new category of vehicle that enables novel possibilities in factories. Applications, such as emergency assistance, surveillance or rapid point-to-point delivery can be highly optimized with these vehicles.
- Autonomous robots [26]: robots have been extensively adopted in industry since commercial variants have been available. Nevertheless, early iterations of robotics technologies were limited in the number of tasks that they could perform and depended strongly on operators programming them correctly. Currently, AI and ML, along with Simultaneous Location and Mapping (SLAM) and navigation technologies, are enabling novel functionalities on robots that are much more autonomous and perform tasks that were previously reserved for workers.
- Connected workers solutions [27]: the development of consumer electronics in the last years has had a higher impact in the professional area. Gadgets, such as Augmented Reality (AR) glasses, tablets, haptic interfaces and sensors have shown a productivity boost in factories.

All of these technologies rely on wireless connectivity. Some of them, such as production lines, are already mature technologies where a mobility component is added in the Industry 4.0 paradigm. In that cases, wired connections need to be changed to wireless [28].

### 2.2. 5G Multi-Connectivity Overview

Multi-connectivity in 5G New Radio (NR) inherits from the Long Term Evolution (LTE) Dual Connectivity (DC) concept. LTE DC was first specified in Release 12 [29] and allows UE to simultaneously send/receive data from different evolved NodeBs (eNBs). The data split is performed at the Packet Data Convergence Protocol (PDCP) layer of the transmitting eNB. At the receiving side, the information is decoded from lower layers, and it is combined at the PDCP layer on the receiver. This process allows boosting the throughput [30].

In Release 15, multi-RAT DC was specified for DC operation with NR and LTE nodes [31]. Not only data split but also PD at the PDCP layer is introduced. PD allows the same packet to be transmitted by different nodes, thus, improving the reliability. The nodes are commonly known as the Master Node (MN) and Secondary Node (SN) and are interconnected via a Xn interface. MN is in charge of activating/deactivating PD via Radio Resource Control (RRC) signaling [12]. If the different links are spatially uncorrelated, transmitting the duplicate packet can compensate poor channel conditions. This is very important in industrial scenarios, where the fundamental problems are interference and multipath propagation, due to the presence of concrete walls and large metallic machinery and structures.

Moreover, NR-NR DC for standalone deployments was standardized in Release 16 [31], in which a UE is connected to one gNB that acts as a MN and another gNB that acts as a SN. In this paper, NR-NR DC with the PD approach is assumed for the downlink direction.

#### Packet Duplication for URLLC

PD is a multi-connectivity solution that improves reliability by increasing redundancy of the transmission. When PD is activated, the PDCP entity in the MN is responsible for PD, whereas the PDCP entity in the receiver is responsible for detecting and removing duplicated packets. The PDCP entity duplicates the packet data unit (PDU) to avoid twice performing functions, such as ciphering, header compression, integrity protection etc. This PDCP PDU has the same sequence number in both.

Then, the packet is forwarded by MN to the SN via Xn-U interface for transmission to the UE. The packet will undergo through independent Radio Link Control (RLC), Medium Access Control (MAC) and physical layer processing at each gNB. This implies that the packet can be transmitted at different time intervals and over different frequency resources and that physical transmission aspects, such as beamforming, MCS, ACK/NACK signaling and the Hybrid Automatic Repeat Request (HARQ) mechanism, are independent.

**Figure 1:**
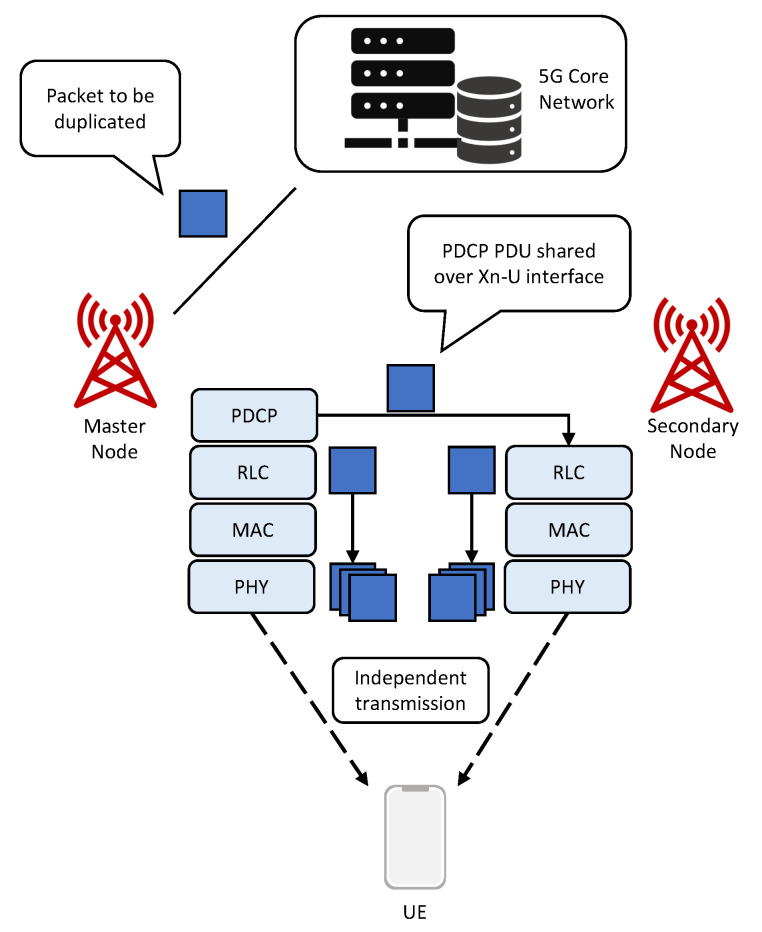
A downlink packet duplication scheme in a NR-NR DC scenario.

On the receiver side, multiple copies of the packet are received, and the UE will forward the first successfully received packet to the higher layers and remove duplicated packets received later, based on the PDCP sequence number. Figure 1 shows a 5G NR-NR PD scheme for downlink transmission.

As PD improves reliability, it becomes a suitable mechanism for URLLC, which demands a higher reliability and low latency. Not only to improve reliability but also to reduce latency, as independent transmissions are performed by two different gNBs, as mentioned before. In this case, latency reduction will be dependent on the best link. Reliability and latency for URLLC are dependent on each other, that is, high reliability is consequential only if packets are received within the latency constraint. As a consequence, PD may be used to improve both the reliability and latency.

Nevertheless, PD operates at the cost of wasting resources on SN. Thus, it is not efficient to always send packets duplicated, as there could be situations where the conditions are fulfilled by the primary node. For that reason, it is important to provide a dynamic mechanism for MN that controls the activation/deactivation of PD for URLLC devices, in order to reduce the resource costs at SN, which may affect other UEs attached on SN.

In this paper, we propose the use of a ML predictor, which, based on channel conditions, predicts the E2E latency for URLLC devices in order to activate/deactivate duplication for a packet transmission.

## 3. Proposal

This section describes the proposed solution in order to assess reliability for URLLC communications by using a dynamic packet duplication algorithm based on ML to reduce the resource consumption.

### 3.1. System Description

The objective of the system described in this paper is to provide a prediction of the E2E latency to assess the need for using PD solution. The proposed system provides an estimator for downlink transmissions that is trained offline in a server running in the network edge to reduce the computing workload and memory requirements at the master node.

The end devices may be installed in production line elements, AGVs, drones etc. These terminals may need to receive messages with a certain guarantee of latency and reliability. The reliability and latency can be improved by using multi-connectivity, that is, duplicating the packet via two paths, with the cost of dramatically increasing the amount of radio resources spent per transmission.

An alternative way, as presented in this paper, is to first predict whether the network will be able to provide that guarantee without using PD. The MN, right before transmitting, can estimate if a regular transmission, that is, without extra resources, will be sufficient or if PD technique needs to be established. When URLLC devices are not involved in any processor-intensive task, they will measure the latency and report the measurements along with the KPIs to the system where a ML method will update the estimation model.

**Figure 2:**
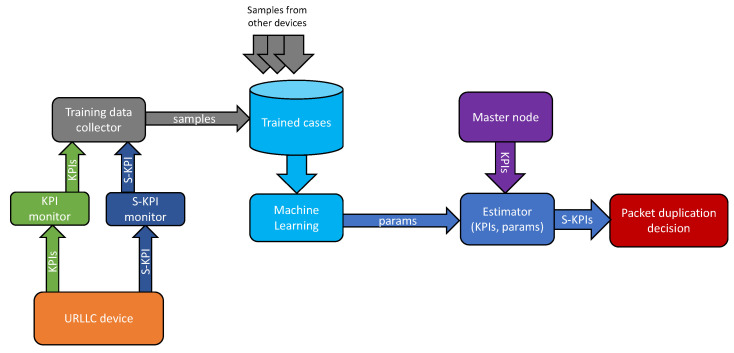
Block diagram of the system.

Figure 2 shows the overall architecture of the system. There are three main domains: the device, which contains the sample collection modules; the server, which runs the ML algorithm and stores a dataset with solved trained cases; and the MN, which contains the estimator. The device needs to receive URLLC messages, and therefore KPIs and S-KPI are forwarded to the server, which runs the ML algorithm and is located at the network edge with computational resources and access to a non-restrictive power source.

The server will collect the data gathered by the devices and generate the ML parameters (prediction model) for the estimator. Finally, the MN uses the estimator to predict the E2E latency of URLLC devices. Based on the prediction, MN will activate/deactivate PD for the current packet transmission.

The functions of the different modules of the system are explained below:

- KPI monitor: collects the KPIs from the radio interface at regular intervals.
- S-KPI monitor: reads the information from the URLLC device and measures the latency.
- Training data collector: joins the data generated by the KPI monitor and the S-KPI monitor. The data joined is used as input to train the ML model.
- Estimator: performs the task of estimating the S-KPI. The primary inputs are the current KPIs (such as SINR, MCS, HARQ feedback etc.) as measured by the MN. The output is the estimation of the E2E latency. This module also has a secondary input that consist in the estimation model extracted from the ML.

### 3.2. KPI to S-KPI Mapping

In cellular networks, performance monitoring (PM) indicators are collected from different points of the network: gNBs, radio interfaces, core network etc. UE traces can also be collected, representing the radio PM indicators collected by the terminals. The PMs are sent to a centralized location where they are analyzed.

Monitoring the network provides many alternatives for detection of problems, analysis of the performance, among others. Nevertheless, these KPIs contains information from lower layers of the network, but not on the performance at application layer in the UEs. Since the development of 5G is centered on the E2E Quality of Service (QoS), this approach has gained importance in recent years. S-KPIs [17] measure these magnitudes. S-KPIs are specific to the final application; that is, for a video transmission, relevant S-KPIs include the average buffer size or the number of stalling, while, for delay-sensitive applications, the main S-KPI is the E2E latency.

The main inconvenience of S-KPI is that they are difficult to measure. They require special procedures at the application layer of the device. The E2E latency can only be measured a posteriori, thus, becoming useless to decide whether a special transmission, such as PD can be performed at a given time.

To address this, a KPI to S-KPI estimator can be used [19]. This technique allows an estimation of the S-KPI based on the available KPIs, which are easy to obtain. Since the KPIs that can be obtained are only a subset of the variables that influence the value of the S-KPI, the estimator will always have a margin for error, and the formula for the mapping will not be trivial. In [19], the approach for creating such estimator is with ML techniques.

In this paper, the hypothesis is that, through this mapping, the E2E latency can be obtained a priori by monitoring the following KPIs:

- Signal to Interference plus Noise Ratio (SINR): includes all the usable signals in the computation. It is used by some vendors to better determine the CQI to adapt the modulation.
- Modulation index: indicates the modulation index used from the table of the MCS when performing a packet transmission. A higher index selects a more efficient modulation, with a higher spectral efficiency and code rate. Otherwise, a lower modulation index selects a more robust modulation, with a lower spectral efficiency and code rate.
- Reception Success: indicates if a packet has been decoded successfully at the receiver or not. This is used to determine if a packet has suffered a HARQ retransmission, since the NACK message is indicated by the receiver to the base station.

The hypothesis assumes that the RAN network is the main contributor to the variable part of the latency. This is due to the jitter being higher at Radio Access Technologies (RATs) [32] as opposed to the wired networks present in the trunk [33]. When using MEC, the processing is done with the gNB or after a negligible network path, and thus the trunk network component is canceled completely. Latency does not depend directly on the measured KPIs but instead on factors, such as the network load. Nevertheless, in this paper, it is assumed that these KPIs represent the overall status of the RAN connection.

Since the relations between the measured KPIs and the factors that determine the latency are complex, ML is commonly used to find and exploit such relations. In this paper, ML generates the prediction model that encompasses the complex relations among the cited KPIs and the latency.

### 3.3. Random Forests

In this paper, random forests [34] were selected for the implementation of the ML part of the system. The choice of this method is based on the computation simplicity once the model has been trained, since fast prediction for URLLC services must be performed.

Random forests are an ensemble method commonly used to resolve several types of ML learning problems, such as classification and regression. A random forest consists of a set of decision trees. Each decision tree takes the input to the forest and returns an estimated value. The structure of the tree is created in the training process. On each tree, a decision is performed by comparing the input with a threshold. Based on the output of the comparison, a new comparison is performed with a different input and threshold, which determines the prediction of the tree.

**Figure 3:**
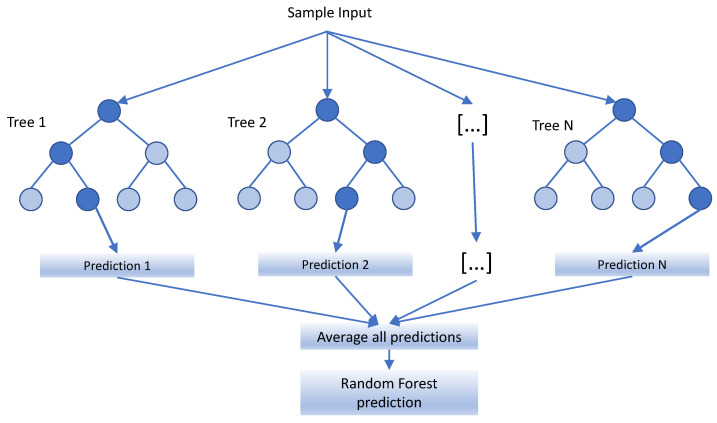
Random forest prediction scheme.

To improve the accuracy, each decision tree output is aggregated. For regression, the aggregation method is typically the average of the output of all the trees. A summarized scheme of the random forests prediction is shown in Figure 3.

Moreover, several parameters must be adjusted before starting the execution, related to the performance of both the decision trees and the complete set that builds the forest. Some of the most important configuration parameters are the following:

- Number of decision trees: this establishes the number of trees that constitutes the forest. This must be chosen in relation to the input dataset to avoid overhead.
- Bootstrap: this parameter decides how each tree is built independently. If it is not activated, the complete dataset is used for each tree. Otherwise, the initial dataset is divided into subsets of dataset for each tree.
- Division criterion: this defines the quality of a split according to the condition set in the node. The most used criteria for regression are the squared error and absolute error.
- Maximum leaves per tree: this sets the maximum depth of the tree in the forest.
- Maximum samples to split: this determines the maximum number of samples to consider in order to choose the condition that determines the split.
- Minimum samples to split: this determines the minimum samples needed to consider a new split.

A training method is applied over a set of labeled samples, (vectors with inputs to the system and the expected output) to obtain the trees. The training process [34] consists of randomly selecting a subset of features and a subset of training samples for each tree and then training it using the CART [35] algorithm without pruning. The random selection of samples contributes in avoiding overfitting. Once the model has been trained, an evaluation phase is performed, where only the values of the different inputs are considered, and the algorithm provides the output. Then, the output of the model and the original are compared in order to estimate the accuracy of the model.

In the ML scheme proposed in this paper, the labeled samples are collected in the UEs and transmitted to a server located at the network edge, where different datasets are collected. The collection of different datasets helps the KPI to S-KPI mapping by capturing the effects of contextual variables. The ML process is executed in a server, and the estimator is used by the MN to predict the S-KPI. In particular, the inputs of the algorithm are the SINR, the modulation index and the reception success, as previously indicated in Section 3.2. The output will be the E2E latency experienced by the URLLC devices.

### 3.4. Implementation Considerations

While the measurements in this paper were performed on simulations, it is important to discuss the issues that arise when this implementation is ported to the real world, in a real MN and stock UEs. Specifically, in this subsection, the aspects on data collection requirements, the need for retraining and the hardware and software requirements both on the MN and the UE are discussed.

Regarding the data, the ML algorithm requires data on KPIs and S-KPIs. KPIs are easily collected by the Network Monitoring System (NMS), which is part of the maintenance systems of the MN. The S-KPIs are harder to obtain, since they require the collection of data in the UEs. Therefore, to obtain real values, a measurement campaign must be done, including UEs with the appropriate software for capturing and transmitting the S-KPIs.

Another important aspect is the need for retraining the model to adapt it to changes in the environment. The validity of the model depends on variations in the behavior of the features that are used in it, which determine the relation between the independent and dependent variables. Second-order effects, such as contextual factors that are not included in the model (e.g., geometry of the buildings, weather conditions, power supply variations etc.). These effects are out of the scope of the tests in this paper; however, on real tests, the required frequency of retraining would be an important parameter to study.

To implement the proposed functionality in reality, some hardware and software requirements are posed over both the MN elements and the UEs. Specifically, in the MN, the software for data collection (KPIs and S-KPIs), as well as the ML algorithm and the estimator (described in Figure 2) must be implemented in some element of the network. An important aspect to take into account is the latency of the decision. There are two possible implementations:

- Implement the data collection and ML stages in the network core. The main advantage is the availability of large datasets that add diversity to the final model. Another advantage of this method is that cloud computing resources can be used better. This is even more important when looking ahead to future 6G networks, where network elements in the core network for ML and AI are envisioned.
- Implement everything in the network edge. In this case, to gain diversity, a Federated Learning (FL) mechanism can be used to share model parameters between different agents. FL is the collaborative learning, which trains and updates the model through the joint effort of multiple servers that are deployed in a decentralized manner within the network.

In both of these cases, the estimator will run in the network edge to minimize latency in the decision, the KPI collection will be done in the network edge due to the nature of the task, and the S-KPI collection will be done in the terminals. All these software elements must be supported by the appropriate hardware equipment. While ML is hardware intensive, the estimator has a low demand in resources.

Regarding the UE, the hardware and software requirements are quite low. Specifically, the devices must implement a functionality for capturing S-KPI information and sending it to the MN (to wherever the data collection function is implemented; either in the edge or the core). While the requirement is quite low in terms of hardware and software, it has some complications in the form of privacy and confidentiality issues. Therefore, it is expected that the use of UEs with the appropriate software is limited to a reduced set of devices acting over a predefined period of time.

Ownership of the network will also play a central role for this aspect; if the user (in the case of industry, the owner of the factory) is also the owner of the network equipment where the data is collected (edge or core), then it is likely that the rate of penetration of the S-KPI probes is higher in the installed base of UEs and more stable in time. Therefore, this would also be an advantage of the system architecture where the data collection and machine learning is done in the edge, and the model parameters are then shared with FL.

## 4. Tests

The proposed scheme was tested in a simulated 5G network, using ns-3, which is a free and open-source network simulator that is very popular in research [36]. In particular, the 5G-LENA module [37] was selected to conduct the simulations. This module focuses on the new 3GPP NR specifications and includes numerology support, frequency division multiplexing of numerology and an OFDMA-based scheduler. It also includes beamforming and HARQ feedback implementation.

In this section, the simulation scenario along with the KPI recollection phase to train the model are described.

### 4.1. Simulation Scenario

The scenario consists in an indoor factory, with an area of 4800 m^2^ and a height of 10 meters. The scenario is based on the one proposed in 3GPP 38.901 (Table 7.8-7) [38], which was used to calibrate the indoor factory scenario defined in Release 16. In particular, the Indoor Factory with Dense clutter and High base station (InF-DH) [38] scenario was selected with a clutter height of 6 m and clutter density of 80%.

**Figure 4:**
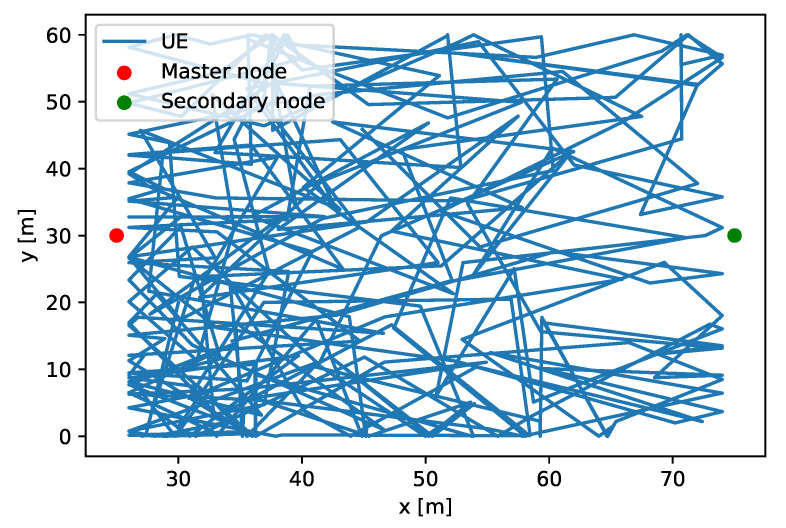
UE movement over the entire scenario.

Attending to the RAN part, there are two picocells with a height of 8 m, which are interconnected via Xn interface, with a base station distance of 50 m. Both gNBs operate with a frequency of 3.7 GHz and a bandwidth of 20 MHz. One transmission/reception omnidirectional antenna was used in both, picocells and UEs, with 23 dBm as downlink transmission power. Figure 4 shows the distribution of the scenario simulated.

The slot length configured is set to 0.25 ms, which corresponds to numerology 2, as defined in the standard [39]; whereas the number of HARQ retransmissions attempts was set to a maximum of 1, due to URLLC latency constraints. Moreover, the link adaptation used at gNB for MCS selection is an error model-based, where the MCS is selected to meet a target transport Block Error Rate (BLER). The MCS table used is Table 1 (up to 64-QAM) from 3GPP 38.214 [40]. The delay for the scheduling procedure was set as following:

- The packet processing from MAC to PHY layer is fixed at two slots. This is a delay between the control/data acquisition from the RLC layer by the MAC layer and the moment at which the data is available to go over the air.
- The transport block decode latency is set to 100 microseconds at UE and gNB. It is a delay between the data acquisition from the air by the PHY layer and the moment at which the data block is available to process at the MAC layer.
- The processing delay needed to decode Downlink Control Information (DCI) and decode downlink data is set to 0 slots.
- The processing delay needed from the end of downlink data reception to the earliest possible start of the corresponding ACK/NACK transmission is set to 1 slot.

On each base station, there are 15 UEs attached, whose positions are fixed and randomly selected at the beginning of the simulation. For each UE, a Constant Bit Rate (CBR) flow of 1 Mbps is sent by the gNB in downlink direction.

On the other hand, there is a URLLC device that is connected to both gNBs simultaneously, one acting as a MN and the other one as a SN. The initial position of the URLLC device is random over the scenario, using a uniform distribution. Then, when the simulation begins, the URLLC device selects a random direction (where all angles are equiprobable, since a uniform distribution is used) over 360 degrees and maintains that direction during 10 s with a speed of 2 m/s.

When the timer expires, a new direction is selected. The URLLC device represents an AGV that is remotely controlled by the network. To do this, the network sends short commands, in this case, UDP packets with a periodicity of 10 ms and size of 64 bytes.

In this paper, a MEC [20] is considered to allocate the URLLC service at the network edge. Thus, the main contribution of the latency comes from the RAN. Table 1 shows the main configuration parameters of the simulations.

### 4.2. KPIs Recollection

The first part of the test consists in obtaining a dataset from URLLC devices—that is, a collection of KPIs in order to train the ML part. To do this, a simulation was performed, where an URLLC device moves from the entire scenario and recollects different KPIs. This movement along with the scenario setup is shown in Figure 4.

Upon a packet reception at UE, it knows the SINR received if the packet was not decoded successfully and the modulation index used in the transmission. These KPIs along with the latency measured are the inputs to train the ML model. Based on these samples, it is possible to train the model when there are sufficient samples.

Once the ML model was trained, the latency predictor is used by the MN. That is, based on the actual conditions, such as SINR, modulation index and HARQ feedback of the previous packet, the estimator predicts the E2E latency. Since URLLC transmission intervals are very short, we assume that the channel conditions are the same between the current packet and the previous (since $\Delta T>T_{coh}$, $T_{coh}\approx \frac{\lambda /2}{v}\approx$ 20 ms is the coherence time during which channel conditions are stable, where λ is the wavelength and *v* is the speed), which is why HARQ feedback from the previous packet was selected as an input.

Based on the output of the estimator, the MN decides whether to duplicate or not the actual packet taking into account a latency threshold. In this case, the latency threshold was set at 2 ms.

## 5. Results and Discussion

This section shows the results obtained by the test explained in Section 4. The first part of the test consists in obtaining a collection of KPIs in order to train the ML part. This is explained in Section 4.2. Once the ML model has been trained, the second part of the test consists in evaluating the accuracy of the predictor, that is, the accuracy of predicting the latency by MN.

Moreover, new simulations upon the scenario presented in the previous section have been done, comparing the latency performance and resources consumption between not using PD (single connection), always duplicating the packet and the dynamic packet duplication based on the prediction model (which has been trained before, as explained in Section 4.2), where the duplication is performed when the latency predicted by the model is higher than a threshold. Upon this test, latency threshold of 2 ms was chosen.

### 5.1. Prediction Results

**Figure 5:**
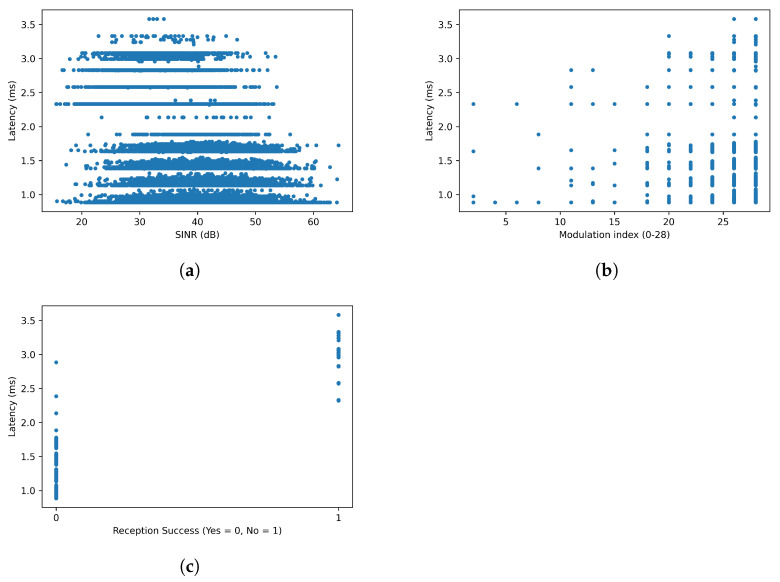
Latency samples. (**a**) SINR, (**b**) Modulation index and (**c**) Reception Success.

This subsection shows the results obtained by the predictor when using the test samples, to measure the accuracy of the predictor. In the test, there are 59,940 samples—packets transmitted by the MN to the URLLC device. Figure 5 shows the distribution of the samples. In general, these distributions show that, as expected, with worse radio conditions, a higher latency is experienced. This is because, with high attenuation, retransmissions at lower layers increase.

The latency with SINR at intermediate values seems to be below or above 2 ms. The increase on the latency is due to HARQ retransmissions that occur. These retransmissions occur when there is a suddenly drop of the SINR, commonly in industrial scenarios, and the modulation index used is still higher (that drop of the SINR was not expected).

This effect is clearly visible in the latency of the modulation index, where robust modulation decrease the probability of HARQ retransmission. Nevertheless, the higher the modulation index is, the higher the probability of retransmission is, as SINR drops occurs. Finally, the latency when the packet decodification was not successful at the PHY layer at UE (that is, a retransmission needs to be performed) is higher, whereas when the reception was successful when the latency was lower as expected.

Table 2 shows the results of the estimator in the validation part. In particular, the false positive rate, the false negative rate and the success rate are given for the predictor for latency. As mentioned above, the latency predictor estimates whether the latency will be higher than 2 ms.

The false positive rate indicates the proportion of times where the actual latency was lower than the threshold but the estimator predicted it would be higher. In this case, the false positive rate is 0.0041%. The false negative rate indicates the proportion of times where the actual latency was higher than the threshold and the estimator failed at predicting this. Ideally, this value should be close to zero. In the results, the latency estimator has a 0.0615% of error rate. The success rate indicates the percent of times where the value of latency was correctly predicted to be either below or above the threshold. In the results, the latency estimator has a 99.9849% of success rate.

### 5.2. Packet Duplication Results

This subsection presents the results obtained when the ML algorithm was trained and the PD approach is used. In particular, a latency comparison between not using PD (single connection), always duplicating the packet (always PD) and the proposed dynamic PD algorithm is performed. Furthermore, for the PD techniques, a comparison of the resource consumption and latency obtained is performed.

**Figure 6:**
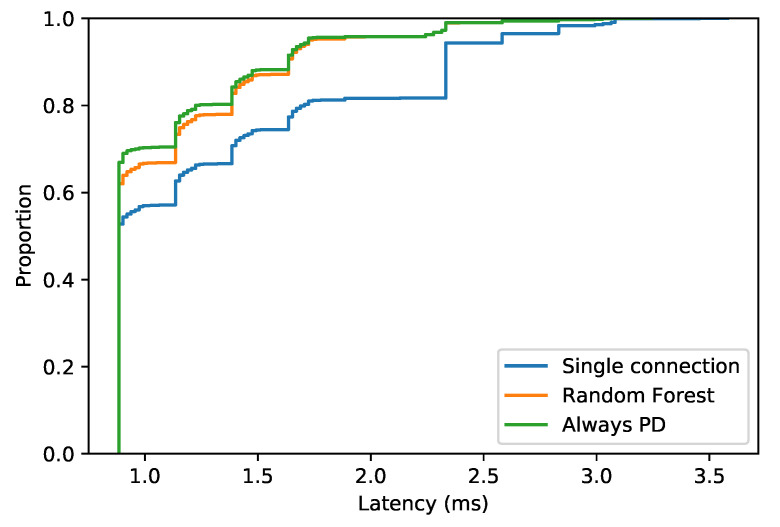
ECDF of the latency received.

Figure 6 shows the empirical cumulative distribution function (ECDF) of the latency obtained when PD is not used (single connection), when always duplicating the packet and when activating PD based on the latency prediction using Random Forest, which is the system proposed in this paper. First of all, as it can be seen, the random forest and always PD distributions are similar, and they converge at 1.75 ms. The probability of receiving lower values of latency (below 1 ms) is 70%, 62% and 55% for always PD, random forest and single connection, respectively. There is a remarkable difference on the probability of receiving a latency below 2 ms between both PD methods and by transmitting via single connection. In this case, there is a gap of 20% on the probability.

The latency below the threshold rate when using PD and not using PD is shown in Table 3, where PD techniques demonstrate that improve the reliability by duplicating the packet incoming for the UE.

**Figure 7:**
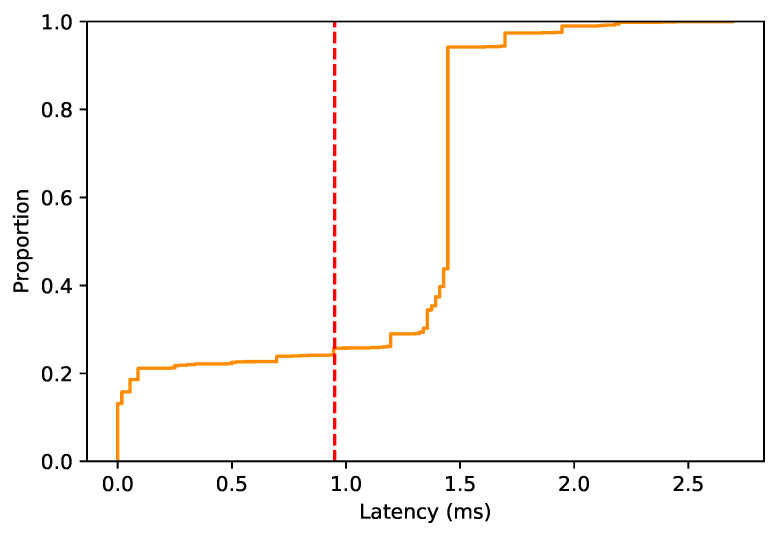
ECDF of the latency gain when the predictor activates PD.

The latency gain distribution when PD is activated by the prediction model is shown in Figure 7. The distribution shows that, when the estimator predicts that latency will be higher than the threshold, in general, there is a latency gain compared to sending over a single link. The latency gain helps to improve the reliability of URLLC communications. The vertical dashed line represents the 75% percentile. In this case, the probability of reducing the latency more than 1 ms is 75%. Otherwise, the probability of not reducing the latency so far, that is, between 0.1 and 0.2 ms, is below 20%.

Table 4 shows the results obtained when using a static PD technique, that is, always duplicating the packet transmitted and the results obtained when using PD only when the latency prediction is above the threshold. In particular, the number of packets that have been sent duplicated, the latency below the threshold rate, the average (packet) latency reduction rate and the PD reduction rate.

Taking into account the number of packets duplicated, it is remarkable that, when using random forest, the number of packets duplicated is lower, specifically, the PD reduction rate is 81.0211%. Both techniques present a similar latency below the threshold rate, with 95.7891% for always PD and 95.7541% when using random forest. That clearly indicates that a dynamic PD is more suitable, guaranteeing the same level of low latency but without wasting extra resources.

The average (packet) latency reduction rate measures the proportion of times where latency was reduced when duplicating the packet. In the results, the average (packet) latency reduction rate is better when duplicating via random forest than always duplicating, obtaining a latency reduction rate of 86.5506% for the system proposed and 25.0917% for a static duplication. The lower rate when always duplicating is due to sending the packet via two path when conditions are favorable at MN—that is, the latency constraint is fulfilled by MN link.

## 6. Conclusions

Many solutions have been proposed for URLLC connectivity in order to achieve high reliability and low latency; however, these typically come at a cost in terms of resource usage. This paper proposes a scheme to predict the E2E latency in order to determine if the PD technique is required for downlink transmissions (dynamic PD) and, thus, reduce the resource wasting.

The proposed algorithm uses a ML approach, where an estimator is running in the MN. The algorithm was implemented and tested in a 5G simulator, showing high accuracy for determining whether or not to activate PD for a packet transmission.

Moreover, the PD technique was evaluated using the predictor (once the model is already trained). The results obtained show that the proposed dynamic PD based on the E2E latency prediction is more efficient than always duplicating, obtaining a high PD reduction rate (81%) and maintaining the same level of latency below the threshold.

In addition, when activating PD based on the predictor, the latency reduction is higher than when using always duplicating technique, that is, there are so many unnecessary packets duplicated, where the latency constraint can be fulfilled by a single connection. A comparison between PD techniques and a single connection was performed, where PD techniques demonstrate that help to achieve URLLC latency constraint.

As a future line of work, some of the implementation aspects will be studied. A major milestone toward the real implementation of the system will be the study of FL for the creation and enrichment of a model. Another important aspect will be the study of the required model update frequency, which may also be reduced due to FL.

## Figures and Tables

**Table 1:** The main configuration parameters.

Parameter	Value
Channel and propagation loss model	3GPP 38.901
System bandwidth	20 MHz
Center frequency	3.7 GHz
Numerology	2
Scenario	InF-DH
Transmission direction	Downlink
Modulation	Adaptive
Scheduler	Round-Robin
UE height	1.5 m
gNB height	8 m
Transmission power	23 dBm
Xn interface delay	100 μs
MAC to PHY delay	2 slots
Transport block decode latency	100 μs
HARQ feedback delay	1 slot
HARQ retranmission attempts	1
Packet size	64 bytes
Packet interval	10 ms

**Table 2:** Prediction results.

S-KPI	False Positive Rate	False Negative Rate	Success Rate
Latency	0.0041%	0.0615%	99.9849%

**Table 3:** Latency below the threshold rate for the different techniques.

Technique	Latency below Threshold Rate
Single connection	81.6549%
Always PD	95.7891%
PD via Random Forest	95.7541%

**Table 4:** Comparison results between static and dynamic PD.

PD Technique	Number of Packets Duplicated	Latency below Threshold Rate	Average (Packet) Latency Reduction Rate	PD Reduction
Always PD PD via	59,940	95.7891%	25.0917%	Not applicable
Random Forest	11,376	95.7541%	86.5506%	81.0211%

## Data Availability

Not applicable.

## References

[1] Lasi H., Fettke P., Kemper H.G., Feld T., Hoffmann M. (2014). Industry 4.0. Bus. Inf. Syst. Eng..

[2] 3GPP TR 38.913, Study on Scenarios and Requirements for Next Generation Access Technologies; V14.3.0, Rel-14. https://portal.3gpp.org/desktopmodules/Specifications/SpecificationDetails.aspx?specificationId=2996.

[3] 5G Americas New Services & Applications with 5G Ultra-Reliable Low Latency Communications; 5G Americas White Paper, November 2018. https://www.5gamericas.org/wp-content/uploads/2019/07/5G_Americas_URLLLC_White_Paper_Final__updateJW.pdf.

[4] Zaidi A.A., Baldemair R., Tullberg H., Bjorkegren H., Sundstrom L., Medbo J., Kilinc C., Da Silva I. (2016). Waveform and Numerology to Support 5G Services and Requirements. IEEE Commun. Mag..

[5] Segura D., Khatib E.J., Munilla J., Barco R. (2021). 5G Numerologies Assessment for URLLC in Industrial Communications. Sensors.

[6] Pedersen K., Pocovi G., Steiner J., Maeder A. (2018). Agile 5G Scheduler for Improved E2E Performance and Flexibility for Different Network Implementations. IEEE Commun. Mag..

[7] Jacobsen T., Abreu R., Berardinelli G., Pedersen K., Mogensen P., Kovacs I.Z., Madsen T.K. System Level Analysis of Uplink Grant-Free Transmission for URLLC. Proceedings of the 2017 IEEE Globecom Workshops (GC Wkshps).

[8] Khatib E.J., Wassie D.A., Berardinelli G., Rodriguez I., Mogensen P. Multi-Connectivity for Ultra-Reliable Communication in Industrial Scenarios. Proceedings of the 2019 IEEE 89th Vehicular Technology Conference (VTC2019-Spring).

[9] Mahmood N.H., Lopez M., Laselva D., Pedersen K., Berardinelli G. Reliability Oriented Dual Connectivity for URLLC services in 5G New Radio. Proceedings of the 2018 15th International Symposium on Wireless Communication Systems (ISWCS).

[10] Rayavarapu S.M., Amuru S.D., Kiran K. Dynamic Control of Packet Duplication in 5G-NR Dual Connectivity Architecture. Proceedings of the 2020 International Conference on COMmunication Systems NETworkS (COMSNETS).

[11] Huang Y., Hou Y.T., Lou W. A Deep-Learning-based Link Adaptation Design for eMBB/URLLC Multiplexing in 5G NR. Proceedings of the 2021 IEEE Conference on Computer Communications (INFOCOM).

[12] Rao J., Vrzic S. (2018). Packet Duplication for URLLC in 5G: Architectural Enhancements and Performance Analysis. IEEE Netw..

[13] Belogaev A., Khorov E., Krasilov A., Shmelkin D., Tang S. Conservative Link Adaptation for Ultra Reliable Low Latency Communications. Proceedings of the 2019 IEEE International Black Sea Conference on Communications and Networking (BlackSeaCom).

[14] Khan J., Jacob L. Link Adaptation for Multi-connectivity Enabled 5G URLLC: Challenges and Solutions. Proceedings of the 2021 International Conference on COMmunication Systems and NETworkS (COMSNETS).

[15] Centenaro M., Laselva D., Steiner J., Pedersen K., Mogensen P. Resource-Efficient Dual Connectivity for Ultra-Reliable Low-Latency Communication. Proceedings of the 2020 IEEE 91st Vehicular Technology Conference (VTC2020-Spring).

[16] Zhao Q., Paris S., Veijalainen T., Ali S. Hierarchical Multi-Objective Deep Reinforcement Learning for Packet Duplication in Multi-Connectivity for URLLC. Proceedings of the 2021 Joint European Conference on Networks and Communications 6G Summit (EuCNC/6G Summit).

[17] Lorca J., Solana B., Barco R., Herrera-Garcia A., Palacios D., Fortes S., Demestichas P., Kosmatos E., Georgakopoulos A., Stavroulaki V. (2017). Deliverable D2.1: Scenarios, KPIs, Use Cases and Baseline System Evaluation. Technical Report, E2E-Aware Optimizations and Advancements for Network Edge of 5G New Radio (ONE5G). https://one5g.eu/wp-content/uploads/2017/12/ONE5G_D2.1_finalversion.pdf.

[18] Aijaz A. (2019). Packet Duplication in Dual Connectivity Enabled 5G Wireless Networks: Overview and Challenges. IEEE Commun. Stand. Mag..

[19] Herrera-Garcia A., Fortes S., Baena E., Mendoza J., Baena C., Barco R. (2019). Modeling of Key Quality Indicators for End-to-End Network Management: Preparing for 5G. IEEE Veh. Technol. Mag..

[20] Hu Y.C., Patel M., Sabella D., Sprecher N., Young V. (2015). Mobile Edge Computing—A key technology towards 5G. ETSI White Pap..

[21] Hasan S., Ben-David Y., Bittman M., Raghavan B. The Challenges of Scaling WISPs. Proceedings of the 2015 Annual Symposium on Computing for Development (DEV’15). Association for Computing Machinery.

[22] Rostami A. Private 5G Networks for Vertical Industries: Deployment and Operation Models. Proceedings of the 2019 IEEE 2nd 5G World Forum (5GWF).

[23] Weyer S., Schmitt M., Ohmer M., Gorecky D. (2015). Towards Industry 4.0—Standardization as the crucial challenge for highly modular, multi-vendor production systems. IFAC-PapersOnLine.

[24] Mehami J., Nawi M., Zhong R.Y. (2018). Smart automated guided vehicles for manufacturing in the context of Industry 4.0. Procedia Manuf..

[25] Fernández-Caramés T.M., Blanco-Novoa O., Froiz-Míguez I., Fraga-Lamas P. (2019). Towards an Autonomous Industry 4.0 Warehouse: A UAV and Blockchain-Based System for Inventory and Traceability Applications in Big Data-Driven Supply Chain Management. Sensors.

[26] Gonzalez A.G., Alves M.V., Viana G.S., Carvalho L.K., Basilio J.C. (2017). Supervisory control-based navigation architecture: A new framework for autonomous robots in industry 4.0 environments. IEEE Trans. Ind. Inform..

[27] Paelke V. Augmented reality in the smart factory: Supporting workers in an industry 4.0. environment. Proceedings of the 2014 IEEE Emerging Technology and Factory Automation (ETFA).

[28] Mogensen R.S., Rodriguez I., Berardinelli G., Fink A., Marcker R., Markussen S., Raunholt T., Kolding T., Pocovi G., Barbera S. Implementation and Trial Evaluation of a Wireless Manufacturing Execution System for Industry 4.0. Proceedings of the IEEE 90th Vehicular Technology Conference (VTC2019-Fall).

[29] 3GPP TS 36.300, Evolved Universal Terrestrial Radio Access (E-UTRA) and Evolved Universal Terrestrial Radio Access Network (E-UTRAN); Overall Description; Stage 2; V12.10.0, Rel-12. https://portal.3gpp.org/desktopmodules/Specifications/SpecificationDetails.aspx?specificationId=2430.

[30] Rosa C., Pedersen K., Wang H., Michaelsen P.-H., Barbera S., Malkamäki E., Henttonen T., Sébire B. (2016). Dual connectivity for LTE small cell evolution: Functionality and performance aspects. IEEE Commun. Mag..

[31] 3GPP TS 37.340, Evolved Universal Terrestrial Radio Access (E-UTRA) and NR; Multi-Connectivity; Stage 2; V16.0.0, Rel-16. https://portal.3gpp.org/desktopmodules/Specifications/SpecificationDetails.aspx?specificationId=3198.

[32] Kassim M., Rahman R.A., Aziz M.A.A., Idris A., Yusof M.I. Performance analysis of VoIP over 3G and 4G LTE network. Proceedings of the 2017 International Conference on Electrical, Electronics and System Engineering (ICEESE).

[33] Alderisi G., Iannizzotto G., Bello L.L. Towards IEEE 802.1 Ethernet AVB for Advanced Driver Assistance Systems: A preliminary assessment. Proceedings of the 2012 IEEE 17th International Conference on Emerging Technologies Factory Automation (ETFA 2012).

[34] Geurts P., Ernst D., Wehenkel L. (2006). Extremely randomized trees. Mach. Learn..

[35] Breiman L. (2001). Random Forests. Mach. Learn..

[36] NS-3-A Discrete-Event Network Simulator for Internet Systems. https://www.nsnam.org/.

[37] Patriciello N., Lagen S., Bojovic B., Giupponi L. (2019). An E2E simulator for 5G NR networks. Simul. Model. Pract. Theory.

[38] 3GPP TR 38.901, Study on Channel Model for Frequencies from 0.5 to 100 GHz; V16.1.0, Rel-16. https://portal.3gpp.org/desktopmodules/Specifications/SpecificationDetails.aspx?specificationId=3173.

[39] 3GPP TR 21.915, Release Description; Release 15; V15.0.0, Rel-15. https://portal.3gpp.org/desktopmodules/Specifications/SpecificationDetails.aspx?specificationId=3389.

[40] 3GPP TS 38.214, NR; Physical Layer Procedures for Data, Release 16; V16.7.0, Rel-16. https://portal.3gpp.org/desktopmodules/Specifications/SpecificationDetails.aspx?specificationId=3216.

